# Botanical from the Fruits Mesocarp of *Raphia vinifera* Displays Antiproliferative Activity and Is Harmless as Evidenced by Toxicological Assessments

**DOI:** 10.1155/2022/4831261

**Published:** 2022-03-29

**Authors:** Gaëlle S. Nguenang, Armelle T. Mbaveng, Idrios N. Bonsou, Godloves F. Chi, Victor Kuete

**Affiliations:** ^1^Department of Biochemistry, Faculty of Science, University of Dschang, Dschang, Cameroon; ^2^Department of Chemistry, Faculty of Science, University of Buea, Buea, Cameroon

## Abstract

*Raphia vinifera* is widely used to treat several diseases including digestive disorders, dysentery, and genitourinary infections. In this study, the mineral contents, the cytotoxicity, and the toxicological effect of the crude CHCl_3_/MeOH extract (RVM) from the mesocarp of *Raphia vinifera* were evaluated. The mineral contents were evaluated using the method described by the Association of Official Analytical Chemists (AOAC). The cytotoxicity of both extract and chemical compounds from the plants was determined by a resazurin reduction assay (RRA). The toxicological studies were carried out using the experimental procedure of the Organization for Economic Cooperation and Development (OECD). After killing the rats, biochemical, histopathological, and hematological studies were performed. The result indicated that RVM is rich in zinc (6.52 mg/100 g of DM) and sodium (194.5 mg/100 g of DM). RVM had a cytotoxicity effect with IC_50_ values lower than 30 *μ*g/mL in 18/18 cancer cell lines tested. These recorded IC_50_ values were between 12.35 *µ*g/mL (toward CCRF-CEM leukemia cells) and 26.66 *µ*g/mL (toward SKMel-505 BRAF wild-type melanoma cells). Raphvinin 4 displayed good cytotoxicity against MaMel-80aBRAF-V600E homozygous mutant with the IC_50_ of 10.42 *μ*M. RVM was relatively nontoxic to rats, the median lethal dose (DL_50_) being above 5000 mg/kg body weight. However, during the oral administration period extending for 28 days, precautions should be taken due to the increase in urinary creatinine level and decrease in spleen weight in the male rats given the highest dose (1000 mg/kg) of extract. Conclusively, the extract of *Raphia vinifera* is weakly toxic in rats and could be further used in the development of anticancer phytomedicines.

## 1. Introduction

Despite the numerous means to fight against cancer, the number of deaths caused by this disease is increasing significantly in many countries [1]. In 2018, WHO (World Health Organization) recorded 9.6 million deaths and 18.1 million new cases because of cancer [2]. Due to morbidity and mortality that it generates, cancer represents a major health problem both nationally and globally. This pathology becomes increasingly difficult to diagnose and to treat when cancer cells develop resistance mechanisms against the usual chemotherapeutic agents [3]. Regarding the increasing resistance developed by these cells, research for alternative treatments should be performed. The varieties of secondary metabolites contained in medicinal plants are responsible for the pharmacological effects including cytotoxic activity [4].

*Raphia vinifera* (Arecaceae) is a plant from the genus *Raphia*; medicinal properties of different parts of the plants have been demonstrated. Raffia wine from *Raphia vinifera* is rich in lactic acid bacteria [5], which prevents the incidence of diarrhea and promotes the course of the immune response in rats; these probiotic isolates could strengthen the immune system in children [6]. Also, many medicinal plants used in Africa have shown interesting antiproliferative properties against the sensitive and multi-drug-resistant (MDR) cancer cells linked to their secondary metabolites [4, 7]. The boiled solution of apical bud of *Raphia* vinifera is used to treat some diseases like genitourinary infections and gonorrhea in West Cameroon. The leaf is used to fight against poison and for various sexually transmitted diseases and witchcraft [8]. To solve liver problems, the young leaves of this plant are used, and the crushed fruits are poured into water to capture fish easily [9, 10].

Palm has been proven to have minerals like calcium [11]. The *Raphia vinifera* fruit pulp and pericarps were found to contain a high concentration of saponins, alkaloids, and oxalate; a moderate concentration of tannin, flavonoid, and steroid; and a low concentration of phytate, phenol, and glycoside, which are responsible for its therapeutic activity [12]. This plant has provided steroidal saponins [13], which are beneficial in preventing tumors and treating many cancers with high efficiency associated with weak toxicological effect [13]. In addition, saponins are also cytotoxic and act by blocking the cell cycle and could significantly disrupt the mitochondrial membrane potential and selectively upregulate the protein levels of Bax, cytochrome C, and cleaved caspase 3/9 and downregulate the levels of Bcl-2 [14]. The pulp of *Raphia* vinifera contains oil that was extracted and characterized physically and chemically by Igwenyi et al. [15].

Many investigations have demonstrated the ability of medicinal plants in the prevention and treatment of many diseases [4, 16]. However, little information is provided on the toxicological effect of plants on consumers. The research on toxicological effects of medicinal plants and their extract is crucial in the development of drugs and to rise human safety [17]. Many toxicological studies have been carried out using *Raphia vinifera* on fish [11], but studies hardly describe the biochemical toxicity of this plant on rats. This investigation was therefore carried out to evaluate antiproliferative potential of *Raphia vinifera* extract and its constituents, as well as the toxicity of the crude extract.

## 2. Material and Methods

### 2.1. Chemicals and Preparation of the Extract

The phytochemicals used were (25*R*)-spirost-5-ene-3*β*, 22*β*-3-O-*β*-D-glucopyranosyl(1 ⟶ 2)-O-*α*-L-rhamnopyranoside (1); (25S)-26-O-(*β*-D-galactopyranosyl)-furost-5-ene-3*β*, 22*α*, 26-trihydroxy-3-O-*β*-D-glucopyranosyl-(1 ⟶ 2)-*α*-L-rhamnopyranoside or raphvinin 1 (2); (25*R*)-26-O-(*β*-D-galactopyranosyl)-furost-5-ene-3*β*, 22*α*, 26-trihydroxy-3-O-*β*-D-glucopyranosyl-(1 ⟶ 2)-[*α*-L-rhamnopyranosyl-(1 ⟶ 3)]-*β*-D-glucopyranoside or raphvinin 2 (**3**); (25*R*)-26-O-[*β*-D-glucopyranosyl-(1 ⟶ 4)]-*β*-D-galactopyranosyl)-furost-5-ene-3*β*, 26-dihydroxy-22*α*-methoxy-3-O-*β*-D-glucopyranosyl -(1 ⟶ 2)-[*α*-L-rhamnopyranosyl-(1 ⟶ 2)] -*β*-D-lucopyranosyl-(1 ⟶ 4)-*β*-D-glucopyranoside or raphvinin 3 (4); diosgenin (5); diosgenin-3-O-*β*-D-glucopyranoside or (22*R*, 25*R*)-3*β*-spirost-5-ene-3-O-*β*-D-glucopyranoside or trillin (6); deltonin (7); 26-O-*β*-D-glucopyranosyl-(22*R*,25*R*)-3*β*, 22, 26-trihydroxyfurost-5-ene-3-O-*β*-D-glucopyranoside (**8**); and sitosterol (**9**). The NMR spectra and the chemical shifts of compounds 1–9 are provided in the Supplementary file (S1to S18). They are isolated from *Raphia vinifera* fruits collected in Bambili, Northwest Region of Cameroon on April 2016, and identified at the Cameroon National Herbarium (voucher number: 38374/HNC) as previously reported [18]. Doxorubicin 98.0% (Sigma-Aldrich) (Munich, Germany) comes from the Medical Center of the Johannes Gutenberg University (Mainz, Germany) and is dissolved in phosphate buffer saline (PBS; Invitrogen, Eggenstein, Germany) at 10 mM. The fruits of *Raphia vinifera* were dried and powdered. This powder (1 kg) was thereafter macerated in CHCl_3_/MeOH (5 L) in the proportions 1 : 1 at room temperature. After 2 days, the extract obtained was filtered with Whatman filter paper (No. 1) and rotary evaporator (Buchi R-200) was used to concentrate the filtrate at 40°C. The crude extract was assembled in sterile flask and dried by oven (40°C) until the solvent completely evaporated.

### 2.2. Cell Cultures and Origins

18 cancer cell lines and normal hepatocyte AML12 were used in the present study. *Cancer* cell lines such as drug-sensitive leukemia CCRF-CEM and multidrug-resistant P-glycoprotein-overexpressing subline CEM/ADR5000 cells [19–21], breast cancer MDA-MB-231-*pcDNA* cells and its resistant subline MDA-MB-231-*BCRP* clone 23 cells [22], colon cancer HCT116 *p53*^*+/+*^ cells and its knockout clone HCT116 p53^−/−^, glioblastoma U87MG cells, and its resistant subline U87MG.Δ*EGFR* [7, 23, 24]. The maintenance of HepG2 cells and AML12 hepatocytes was also published [25]. The CC531 rat colon carcinoma cells, B16-F1, B16-F10, A2058, SK-Mel505, MaMel-80a, MV3, SkMel-28, and Mel-2A, were previously reported [26–30].

### 2.3. Experimental Animals

For the toxicological studies, adult *Wista*r rats (8 to 9 weeks old) of the 2 sexes were selected. To ensure their growth, the animals engaged in the animal house received food daily and tap water. They were maintained at standard laboratory conditions of regular 12 h light/12 h dark cycle. This work was carried out with respect to the well-being of rats like the Institutional Ethical Review Committee of the University of Dschang Cameroon recommended.

### 2.4. Determination of the Mineral Contents

Mineral contents (Ca, P, Mg, Fe, Na, Zn, and K) were determined by the extract using the AOAC method [31]. *Raphia* vinifera fruit extract was introduced into a porcelain crucible and calcinated at 450°C for 2 hrs. The contents of potassium (K), magnesium (Mg), calcium (Ca), sodium (Na), iron (Fe), zinc (Zn), and phosphorus (P) were determined colorimetrically by UV-visible spectrophotometer (Technel 752 P), according to AOAC procedure. Mineral contents of the sample were determined from calibration curves of standard minerals. All minerals were analyzed in duplicate.

### 2.5. Cytotoxicity Assay

Different types of human cancer cell lines were used in this study. The resazurin reduction assay (RRA) as previously described [24, 32] with similar experimental conditions to those reported earlier [26–30] was used to measure the cell cytotoxicity. Fluorescence was measured on an Infinite M2000 Pro™ plate reader (Tecan, Germany) using an excitation wavelength of 544 nm and an emission wavelength of 590 nm. The viability was determined based on a comparison with untreated cells. The values representing the sample's concentrations required to inhibit 50% of cell proliferation (IC_50_) were calculated from a calibration curve by linear regression using Microsoft Excel 2013 [33, 34].

### 2.6. Acute Toxicity Study in Rats

This test was realized under the OECD guidelines [35]. We followed the methods described by Nguenang [36]. Three adult female rats (8–9 weeks) were treated orally with one dose of extract (5000 mg/kg), after 12 hrs of fasting. These rats were individually and frequently observed to check any signs of toxicity during the first day; observation was continued daily for a total of 14 days of the experiment. The body weight of animals on the 15^th^ day was measured. Subsequently, they were anesthetized through intraperitoneal injection with a solution containing diazepam and ketamine (0.2/0.1 ml per 100 grams of the animal), the vital organs such as lung, spleen, heart, kidneys, and liver were removed and weighed, and the macroscopic examinations were performed on those organs. The relative organ weight was determined.

### 2.7. Subchronic Toxicological Study

#### 2.7.1. Treatments

This study was performed under the protocol of the OECD Guidelines [37]. We followed the methods described by Nguenang [36]. Thirty-two *Wistar* rats (16 males and 16 females) aged from 08 to 09 weeks were distributed in 4 groups of 4 rats per group. The groups treated received the doses of 250, 500, and 1000 mg/kg b.w. of extract, while the control group received only distilled water during 28 days of treatment. These rats were individually and frequently observed to check any signs of toxicity. The body weights of all animals were measured after every four days during the experimental period. All animals were weighed on the 29^th^ day, and subsequently, they were anesthetized with solution containing diazepam and ketamine (0.2/0.1 ml per 100 grams of the animal). Blood samples were collected into EDTA and nonheparinized tubes for the measurement of hematological and biochemical parameters, respectively. The organs such as heart, lung, liver, kidneys, and spleen were weighed, and one part of each organ was conserved in the solution of formalin (10%) for histopathological examination. The relative organ weight was determined.

#### 2.7.2. Evaluation of Hematological Parameters

To determine these parameters, the blood sample of rats was collected in the EDTA tubes after the kill. The hematological analysis was performed using an automated analyzer hematology (QBC Autoread plus, United Kingdom). The hematological parameters analyzed included white blood cells (WBCs), hemoglobin (Hb), red blood cells (RBCs), hematocrit (HCT), mean corpuscular volume (MCV), lymphocytes (LYMs), mean corpuscular hemoglobin concentration (MCHC), platelets (PLTs), monocytes, granulocytes, mean corpuscular hemoglobin (MCH), and mean platelet volume (MPV).

#### 2.7.3. Evaluation of Biochemical Parameters

The blood collected in dry tubes was centrifuged at 3000 rpm for 15 min to obtain the serum. The biochemical parameters measured were as follows: total serum protein (TP), alanine aminotransferase (ALT), aspartate aminotransferase (AST), serum creatinine (CREA), alkaline phosphatase (ALP), high-density lipoprotein-cholesterol (HDL-C), total cholesterol (TC), serum urea (UREA), low-density lipoprotein-cholesterol (LDL-C), and triglycerides (TG).

#### 2.7.4. Histopathological Examination

After killing the rats, kidneys and liver were removed and cleaned in saline solution. The parts of these organs were collected for histological studies. These tissues were fixed in formalin (10%) during at least 24 h, dehydrated in a graded series of ethanol (80–100°), and enclosed (embedded) in paraffin. Thereafter, 5-*µ*m sections were prepared using a microtome and stained with hematoxylin-eosin before the microscopic examination. The microscopic features of the animal's (male and female) organs-treated groups were compared with the control group [36, 38].

### 2.8. Statistical Analysis

The data are expressed as mean ± standard deviation (SD). These results have been submitted to the analysis of variance (ANOVA) at one factor according to the general linear model. Statistical analysis was done using version 21 of the IBM-SPSS statistical program, and statistical comparisons were made using the test of Waller Duncan for the subchronic toxicity at the 5% probability level.

## 3. Results

### 3.1. Mineral Content in Extract

The mineral composition of RVM is presented in Table 1. The data obtained from the mineral levels showed that RVM contains more zinc (6.52 mg/100 g of DM) and sodium (194.5 mg/100 g of DM) and less potassium (575.4 mg/100 g of DM), calcium (536.5 mg/100 g of dry matter), phosphorus (277.49 mg/100 g of DM), iron (5.28 mg/100 g of DM), and magnesium (133.67 mg/100 g of DM).

### 3.2. Cytotoxicity of Extract

**Figure 1:**
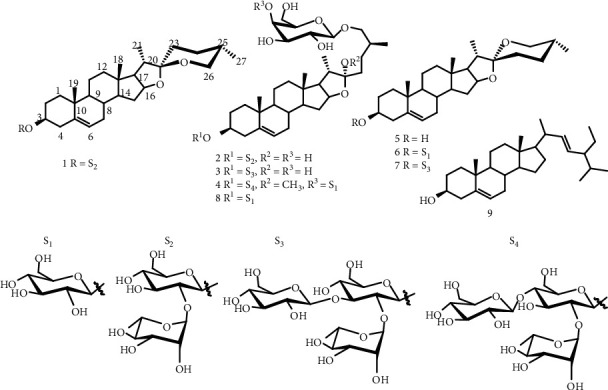
Chemical structures of the tested compounds. (25*R*)-spirost-5-ene-3*β*, 22*β*-3-O-*β*-D-glucopyranosyl(1 ⟶ 2)-O-*α*-L-rhamnopyranoside (1); raphvinin 1 (2); raphvinin 2 (3); raphvinin 3 (4); diosgenin (5); diosgenin-3-O-*β*-D-glucopyranoside or (22*R*, 25*R*)-3*β*-spirost-5-ene-3-O-*β*-D-glucopyranoside or trillin (6); deltonin (7); 26-O-*β*-D-glucopyranosyl-(22*R*,25*R*)-3*β*, 22, 26-trihydroxyfurost-5-ene-3-O-*β*-D-glucopyranoside (8); and sitosterol (9).

The RRA was used to evaluate the effects of RVM, compounds 1–9, and doxorubicin on the proliferation of 18 cancer cell lines and normal AML12 hepatocytes (Tables 2 and 3; Figure 1). The degree of resistance (D. R.) was calculated as the ratio of the IC_50_ value of the resistant cell line divided by that of the corresponding parental sensitive cell line. The D. R. lower than 0.9 was defined as hypersensitivity or collateral sensitivity; D. R. around 1 was interpreted as normal sensitivity, while D. R. greater than 1.2 was signified as cross-resistance. The botanical RVM and doxorubicin revealed antiproliferative effects against the 18 cancer cell lines (Tables 2 and 3). The IC_50_ values obtained were from 12.35 *μ*g/mL (towards CCRF-CEM leukemia cells) to 26.66 *μ*g/mL (against SKMel-505 melanoma cells) for RVM and from 0.02 *μ*M (against CCRF-CEM cells) to 9.39 *μ*M (against SKMel-505 melanoma cells) for doxorubicin. Normal sensitivity was achieved with MDA-MB-231-*BCRP* cells (D.R. of 0.96), with HCT116 (*p53−/−*) (D.R. of 1.05) and CEM/ ADR5000 (D.R. of 1.15), respectively, compared with their sensitive congeners MDA-MB-231-pc DNA cells, HCT116 (p53+/+) cells, and CCRF-CEM cells (Table 2). U87MG.*ΔEGFR* cells (D.R. of 1.35) were cross-resistant to extract compared with their respective sensitive counterpart U87MG cells. RVM (selectivity index (S.I.): 2.76) displayed acceptable selectivity to HepG2 cells as compared to normal AML12 hepatocytes (Table 2). In addition to the fact that RVM had recordable values, extract displayed good antiproliferative activity with IC_50_ values below 20 *μ*g/ mL and 30 *μ*g/ mL in 15/18 and 18/18 cancer cell lines, respectively. Compound 4 (IC_50_: 10.42 *μ*M) displayed good cytotoxicity against MaMel-80aBRAF-V600E homozygous mutant; compounds 4 and 1 displayed moderate antiproliferative activity against 8/9 and 3/9 cell lines, respectively. Compounds 3, 7, 8, and 9 were not active (IC_50_ > 100 *µ*M) towards the tested cell lines.

### 3.3. Acute Toxicity of RVM

During this experiment, no animals died among the female rats receiving 5000 mg/kg of RVM. The signs of toxicities were not detected based on the behavior of rats during the observation period (14 days). Therefore, lethal dose (LD_50_) of this extract was estimated greater than 5000 mg/kg in female rats. Tables 4 and 5 represent the body weights (g) and relative organ weights in the female rats during acute toxicity, respectively.

### 3.4. Subchronic Toxicity of RVM

#### 3.4.1. Food Consumption

**Figure 2:**
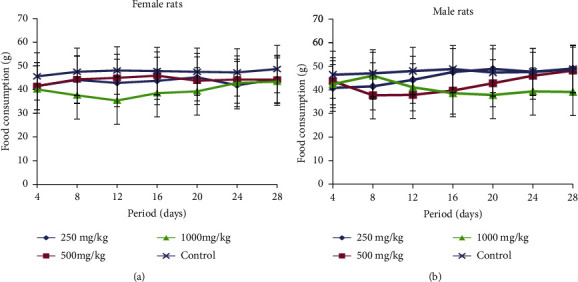
(a). Food consumption changes of female rats treated with RVM during subchronic toxicity study. (b). Food consumption changes of male rats treated with RVM during subchronic toxicity study.

The food consumption changes in both female and male rats treated with different doses (250, 500, and 1000 mg/kg b.w.) of extract are presented in Figures 2(a) and 2(b). During the treatment period, both sexes of rats showed reduction in the food intake compared with the control group. However, the reduction of food consumption was significant from the 16^th^ day of treatment in male rats treated at highest with respect to controls.

#### 3.4.2. Body Weight

**Figure 3:**
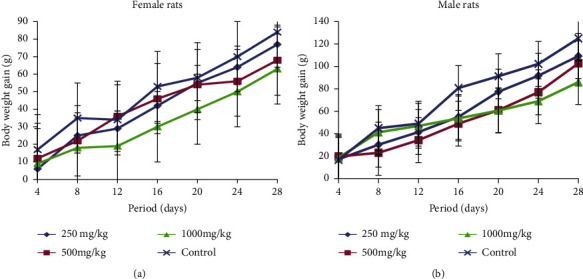
(a). Body weight changes of female rats treated with RVM during subchronic toxicity study. (b) Body weight changes of male rats treated with RVM during subchronic toxicity study.

The body weight gain changes in both female and male rats treated with different doses (250, 500, and 1000 mg/kg b.w.) of extract are represented in Figures 3(a) and 3(b). At all doses during the treatment period, the female and male rats showed a decrease in their body weight compared with the control group. This body weight of treated groups reduced inversely proportional to doses administered with respect to the control group. However, the decrease of body weight in male rats treated at highest dose (1000 mg/ kg) with respect to the control group was significant from the 16^th^ day of treatment.

#### 3.4.3. Organ Weights

Table 6 represents the effect of RVM on organ weights (g) of both female and male rats during subchronic toxicity. The results show that no significant difference (*p* < 0.05) was remarked in organ weights of treated rats with respect to those of the controls. Nevertheless, the spleen weight of male rat was significantly decreased at the dose 1000 mg/kg compared with the control group.

#### 3.4.4. Biochemical Parameters

*(1) Effect of Extract on ALT, AST, ALP Activity, and Total Serum Protein Levels*. The effect of different doses of extract on the activity of transaminases (ALT and AST), total serum protein levels, and alkaline phosphatase levels is shown in Table 7. After repeated administration doses of extract, the results showed that, in female and male rats, the activity of serum total proteins and alkaline phosphatase was significantly reduced at doses 250 and 500 mg/kg compared with the control group. In female rats, the activity of ALT and AST was significantly decreased (*p* < 0.05) at all doses compared with controls. No significant difference was observed in the activity of transaminases at doses 500 and 1000 mg/kg in the male rats. However, in male rats, the significant reduction of these parameters was observed at dose 250 mg/kg compared with the control group.

*(2) Effect of RVM on Level of Urea, Creatinine, and Urinary Protein*. The effects of RVM on the level of serum creatinine, serum urea, and urinary protein are represented in Table 8. Serum and urinary urea level and urinary creatinine level showed a significant reduction in female rats compared with their control group. Urinary urea and serum creatinine levels were significantly reduced in male rats treated at all doses with respect to control groups. However, urinary creatinine level was significantly decreased in rats treated at doses 250 and 500 mg/kg but significantly increased in male rats treated at dose 1000 mg/kg with respect to the control group.

*(3) Effect of RVM on Serum Lipid Profile*. The effect of administration of extract on lipid profile in both female and male rats is represented in Table 9. The HDL cholesterol levels reduced in males treated at three doses compared with the control group. An increment in triglyceride levels (TG) was observed in male rats treated at three doses of extract compared with controls. As compared to the control groups, other parameters measured did not show significant differences.

#### 3.4.5. Hematological Parameters

Table 10 presents the effect of RVM on hematological parameters of the rats treated with RVM. A significant decrease was observed in the level of monocytes, hemoglobin, and hematocrit in the female rats treated with extract at the highest dose compared with controls, while lymphocyte levels significantly reduced in the same group of rats with respect to controls. A significant increase was remarked in the level of platelets in both sexes treated with extract at dose 1000 mg/kg compared with control groups. In male rats, granulocytes were significantly higher in treated animals who received highest dose of extract; the lymphocyte level indicated significant reduction in same group of rats compared with the control group. The significant differences did not show in the rest of hematological parameters measured compared with the control group.

#### 3.4.6. Histopathological Examination

**Figure 4:**
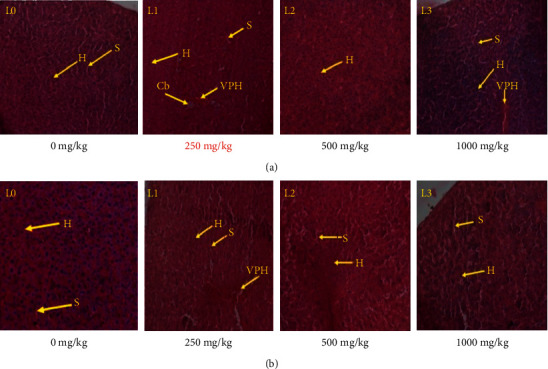
(a). Effect of RVM on liver histopathology in female rats during subchronic toxicity study: (L_0_): control group; (L_1_): 250 mg/kg; (L_2_): 500 mg/kg; and (L_3_): 1000 mg/kg. Indicators: (Cb): bile duct; (VPH): hepatic portal vein; (H): hepatocytes; (S): sinusoid. (b) Effect of RVM on liver histopathology in male rats during subchronic toxicity study: (L_0_): control group; (L_1_): 250 mg/kg; (L_2_): 500 mg/kg; and (L_3_): 1000 mg/kg. Indicators: (VPH): hepatic portal vein; (H): hepatocytes; and (S): sinusoid. The liver photomicrographs presented in the document represent the general appearance observed in at least three of four animals in each group.

**Figure 5:**
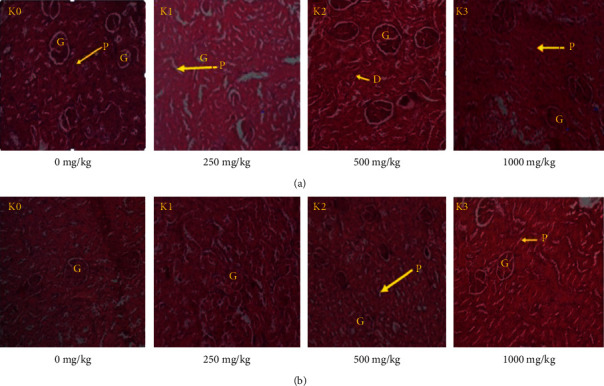
(a) Effect of RVM on kidney histopathology in female rats during subchronic toxicity study: (*k*_0_): control group; (*k*_1_): 250 mg/kg; (*k*_2_): 500 mg/kg; and (*k*_3_): 1000 mg/kg. Indicators: (G): glomerulus; (P): proximal tubule; (D): distal tubule. (b) Effect of RVM on kidney histopathology in male rats during subchronic toxicity study: (*k*_0_): control group; (*k*_1_): 250 mg/kg; (*k*_2_): 500 mg/kg; and (*k*_3_): 1000 mg/kg. Indicators: (G): glomerulus; (P): proximal tubule. The kidney photomicrographs presented in the document represent the general appearance observed in at least three of four animals in each group.

Histopathological examinations were performed on the liver and kidneys to verify whether these organs or tissues had been damaged. No remarkable pathological change was shown on all organs after the microscopic observation compared with the control group. The effect of RVM on liver and kidneys histology in female and male rats during subchronic toxicity study is presented in (Figures 4(a) and 4(b)) and (Figures 5(a) and 5(b)).

## 4. Discussion

Several mineral elements and metabolic products of plant cells are capable to influence the metabolism. These minerals are very important as they have several biological functions, and their deficiency generally leads to nutritional disorders [39]. In this study, zinc (Zn) and sodium (Na) were detected. Zinc plays a vital role in human growth and development. High zinc content was observed in RVM (6.52 mg/100 g DM). The recommended daily dose is between 0.3 and 1 mg/kg in adults [40]. This result is not the same as that of Doungue [41] who obtained 0.88 mg/100 g of DM in *Raphia* (*Raphia hookeri*). This variation of values might be due to the difference in *Raphia* species used.

Medicinal plants are good cytotoxic agents if their IC_50_ value is below 20 *µ*g/mL; phytochemicals are significantly cytotoxic if their IC_50_ < 10 *µ*M and moderately cytotoxic if 10 *µ*M < IC_50_ < 50 *µ*M [42]. Also, according to Suffness and Pezzuto, if the IC_50_ values of plant extracts are lower than or around 30 *µ*g/mL, they deserve to be purified in order to find active components [43]. Hence, plant extract with IC_50_ values lower than 20 *µ*g/mL and 30 *µ*g/mL as obtained in this assay against 15/18 and 18/18 cancer cell lines, respectively. Compound 4 showed IC_50_ equal to 10.42 against MaMel-80aBRAF-V600E homozygous mutant; compounds 4 and 1 showed 10 *µ*M < IC_50_ < 50 *µ*M against 8/9 and 3/9 cell lines, respectively. Regarding criterion of anticancer activities, the plant extracts (RVM), compound 4 (Raphvinin), could therefore be considered as potential cytotoxic drug towards sensitive and resistant phenotypes. Those activities are due to different chemical compounds present in the plant extract. This result is in agreement with those of some authors [18, 26], which have shown that the *Raphia vinifera* compounds have cytotoxicity activity against cancer cell lines. Previous research showed that saponins from *Raphia vinifera* (Progenin III) induced necroptosis, autophagy, and apoptosis in leukemia cells [26]. Zhao et al. have shown that steroidal saponins previously exhibited cytotoxic effects by blocking the *S* phase of interphase [14].

The undesirable effects observed in animals after substance intake predict the toxic effects in humans after its administration [44]. The dosage of markers that ensures the correct functioning of the organism in rats can provide information on the toxicological effect of a substance. For the acute toxicity, the single dose (5000 mg/kg b.w.) of extract administered in rats did not cause animal deaths. Therefore, LD50 was estimated to be greater than 5000 mg/kg since no acute toxicity was detected. The extract has low toxicity when their LD_50_ is between 2000 mg/kg and 5000 mg/kg after oral administration [35]. Some authors had obtained LD_50_ higher than 4000 mg/kg b.w. after administration of the root extract of *Raphia spp.* (*Raphia hookeri*) in same experience [45]. The high safety margin presented by this genus proof its safety for consumers [45].

For the subchronic toxicity study, the rats received 3 doses (250, 500, and 1000 mg/kg) of extract. The results showed that the amount of food intake had a direct effect on animal growth. The significant reductions of food consumption and animal growth were observed in male rats treated at highest dose (1000 mg/kg) from the 16^th^ day of treatment with respect to the control group. The weight loss of animals during this work can be explained by the presence of tannins and saponins (antinutritional substances) in this plant extract. These substances that have the ability to reduce absorption of nutrients in the body [46] would be responsible for the reduction of food consumption, and thus, the reduction in body weight of rats treated at the highest dose of extract. This is agreed with that of Felix and Mello [47] who have reported that tannins showed inhibitory activities on digestive enzymes and decrease the protein quality of foods.

The levels of transaminases and ALP are generally used as biomarkers associated with liver damage [48, 49]. The decrease in serum transaminase, total proteins, and alkaline phosphatase levels at all doses observed in the female rats with respect to control groups could reflect the hepatoprotective activity of secondary metabolite contents in RVM. This result is in agreement with those obtained by Kamga and Russell [9, 12] who have shown that the young leaves of *Raphia vinifera* are used against liver problems. Also, many types of research showed that many flavonoids and saponins present in root, leaf, and epicarp of *Raphia ssp* (*R. hookeri*) have hepatoprotective, antioxidative, anti-inflammatory, and anticancer activities [50–52]. This hepatoprotective activity is proven by histopathological analysis of the liver that revealed no damage in both sexes in rats treated at all doses.

The significant increase of triglyceride levels (TG) was remarked in male rats treated at all doses of extract with respect to their control groups. These could be due to the fact that the extract contains oil, which is rich in triglyceride. This is in accordance with the idea of Igwenyi et al. [15] who have extracted oil from the dried pulp of *Raphia vinifera.* Noubangue et al. [39] have extracted oil from the dried pulp of *Raphia spp* using the maceration method.

Kidney is an organ, which excrete waste product of metabolism outside organism. However, prolonged exposure of the kidney to toxic substances may be altered the renal tubules [53]. The significant reduction of urea and creatinine levels in rats treated with lower doses (250 mg/kg and 500 mg/kg) of extract compared with the control group would be due to the fact that the extract contains secondary metabolism responsible for nephroprotective effect. Some studies have shown that the phenolic compound contents in the extract have nephroprotective activities [52]. However, the apical bud is taken to treat gonorrhea and other genitourinary infections [8]. These results are reinforced by the kidney sections of rats, which present no alteration. The possible kidney malfunction is suspected when the serum levels of creatinine and urea are abnormally high [54]. The increase in urinary creatinine levels in rat treated at dose 1000 mg/kg is due to antinutrient (oxalate) contained in the extract. In fact, the oxalic acid is nocive to the kidney and heart [55] and the symptoms of mild oxalate poisoning include kidney diseases [56].

Hematological components are useful for assessing food's toxicity [57]. The significant increase of lymphocyte levels in female rats treated at highest dose (1000 mg/kg) of extract with respect to their control group was observed since extract contains lactic acid responsible for immunoprotection effect. The investigations performed by some authors showed that raffia wine of *Raphia vinifera* contains lactic acid bacteria [5] that stimulate the immune system of rats [6]. These results agree with those of some authors [58] who have shown that lymphocyte and monocyte levels increase at dose 1000 mg/kg in rat treated with ethanol extracts. The significant increase in blood platelets in both sexes treated at highest dose would indicate thrombocytosis. Moreover, the investigation carried out showed that extracts of *Raphia vinifera* fruit showed an increase in platelet indices counts [58]. The significant reduction of hemoglobin and hematocrit level in female rats treated at highest dose of extract could be because these rats had anemia. Several types of research demonstrate that, when the hemoglobin level is decrease, the patient has anemia [59]. However, investigations carried out by Ogidi et al. [58] showed that methanol and ethanol extracts of RVM showed an increase in red blood cell with respect to control groups. These contradictory results could be explained by the difference in the qualitative or quantitative composition of those two extracts of *Raphia*.

## 5. Conclusion

The aim of this investigation was to evaluate the antiproliferative potential of *Raphia vinifera* extract and its constituents on cancer cells, as well as the toxicity of the crude extract. It also showed that this extract is relatively nontoxic. However, caution should be taken when consuming the extract of the fruit mesocarp of *Raphia vinifera* during 28 days of treatment at highest dose, as it may induce some liver and kidney injuries. In general, *Raphia vinifera* is a safe medicinal plant that deserves further investigation to afford an anticancer phytomedicine.

## Figures and Tables

**Table 1:** Mineral composition of RVM.

Mineral (mg/100 g)	RVM
Calcium	536.5 ± 0.5
Iron	5.28 ± 0.02
Potassium	575.4 ± 0.4
Magnesium	133.67 ± 0.02
Sodium	194.5 ± 0.5
Phosphorus	277.49 ± 0.52
Zinc	6.52 ± 0.02

The table values are presented as mean ± standard deviation.

**Table 2:** Cytotoxicity of RVM and doxorubicin against drug-sensitive cell lines, their resistant counterparts, and normal hepatocytes as determined by RRA.

Cell lines	IC_50_ values (*μ*g/mL) and degrees of resistance ^*∗*^ or selectivity index^*∗∗*^	
	RVM	Doxorubicin
CCRF-CEM	12.35 ± 1.03	0.02 ± 0.00
CEM/ADR5000	14.22 ± 0.98	—
*Degree of resistance* ^*∗*^	(1.15)	Nd
MDA-MB-231-*pcDNA*	17.67 ± 2.01	0.13 ± 0.01
MDA-MB-231-*BCRP*	16.92 ± 0.86	0.79 ± 0.08
*Degree of resistance* ^*∗*^	(0.96)	
HCT116 (*p53*^*+/+*^)	14.56 ± 1.65	0.48 ± 0.06
HCT116 (*p53*^*−/−*^)	15.28 ± 0.67	1.78 ± 0.08
*Degree of resistance* ^*∗*^	(1.05)	
U87MG	13.93 ± 1.16	0.26 ± 0.03
U87MG.Δ*EGFR*	18.76 ± 1.64	0.98 ± 0.07
*Degree of resistance* ^*∗*^	(1.35)	
HepG2	20.13 ± 1.78	4.56 ± 0.48
AML12	56.12 ± 3.77	52.90 ± 4.09
*Selectivity index* ^*∗∗*^	(2.79)	

(^*∗*^): The degree of resistance was determined as the ratio of IC_50_ value in the resistant divided by the IC_50_ in the sensitive cell line; CEM/ADR5000, MDA-MB-231-*BCRP*, HCT116 p53−/−, and U87MG.*ΔEGFR* were used as the corresponding resistant counterpart for CCRF-CEM, MDA-MB-231-pc DNA, HCT116 p53+/+, and U87MG cell lines, respectively; (^*∗∗*^): The selectivity index was determined as the ratio of IC_50_ value in the normal AML12 hepatocytes divided by the IC_50_ in HepG2 hepatocarcinoma cells; nd: not determined.

**Table 3:** Cytotoxicity of RVM, compounds, and doxorubicin against animal cancer cell lines as determined by RRA.

Features and cell lines		Samples, IC_50_ values in *µ*g/mL (extract) or *µ*M (compounds) ± SD						
		RVM	1	2	4	5	6	Doxorubicin
BRAF-V600E homozygous mutant	MaMel-80a	18.92 ± 1.23	33.47 ± 2.06	65.45 ± 7.66	10.42 ± 1.26	—	—	8.66 ± 0.56
	SKMel-28	20.04 ± 0.85	54.28 ± 5.71		14.27 ± 1.10	—	—	2.14 ± 0.12
BRAF-V600E heterozygous mutant	A2058	18.56 ± 2.43	19, 46	56.12 ± 4.29	48.18 ± 5.18	27.06 ± 1.18	38.91 ± 2.78	0.29 ± 0.04
	Mel-2a	23.87 ± 3.19	42.10 ± 3.27	—	45.23 ± 3.29	—	—	6.63 ± 0.41
BRAF wild type	MV3	23.18 ± 1.74	—	—	35.26 ± 2.80	—	—	7.09 ± 0.59
	SKMel-505	26.66 ± 2.19	—	—	43.19 ± 3.72	—	—	9.39 ± 1.01
Rat colon adenocarcinoma	CC531	16.39 ± 0.96	—	58.90 ± 5.25	17.35 ± 3. 1	—	—	0.44 ± 0.23
Murine melanoma	B16-F1	14.98 ± 0.76	—	84.29 ± 6.63	23.19 ± 2.81	—	79.18 ± 5.39	0.22 ± 0.01
	B16-F10	16.55 ± 2.05	—	—	19.89 ± 1.06	—	—	0.24 ± 0.03

(−): IC_50_ values above 100 *µ*M; the IC_50_ values were above 100 *µ*M on all cell lines tested with compounds 3, 7, 8, and 9; ^*∗*^the selectivity index was determined as the ratio of IC_50_ value in the normal AML12 hepatocytes divided by the IC_50_ in other cell lines; (25*R*)-spirost-5-ene-3*β*, 22*β*-3-O-*β*-D-glucopyranosyl(1 ⟶ 2)-O-*α*-L-rhamnopyranoside (1), raphvinin (2), raphvinin 2 (3), raphvinin 3 (4), diosgenin (5), trillin (6), deltonin (7), 26-O-*β*-D-glucopyranosyl-(22*R*,25*R*)-3*β*, 22, 26-trihydroxyfurost-5-ene-3-O-*β*-D-glucopyranoside (8), and sitosterol (9).

**Table 4:** Evolution of body weights (g) of rats treated with RVM during acute toxicity study.

Period (days)	Body weights of female rats (g)		
	Female 1	Female 2	Female 3
1^st^ day	133	128	124
15^th^ day	183	168	166

**Table 5:** Relative organ weights (g) of the female rats treated with RVM during acute toxicity.

Organs	Organ weight of female rats (g)		
	Female 1	Female 2	Female 3
Liver	2.92	2.98	2.80
Kidneys	0.74	0.72	0.66
Lung	0.89	0.73	0.71
Heart	0.32	0.32	0.30
Spleen	0.32	0.33	0.30

**Table 6:** Effect of RVM on organ weights (g) of the rats during subchronic toxicity study.

Sexes	Organs (g)	Control	Extract doses (mg/kg)		
			250	500	1000
Female	Liver	3.12 ± 0.16^a^	2.91 ± 0.17^a^	2.93 ± 0.16^a^	3.06 ± 0.28^a^
	Kidneys	0.65 ± 0.02^a,b^	0.68 ± 0.04^a^	0.61 ± 0.05^b^	0.69 ± 0.03^a^
	Lung	0.57 ± 0.04^a^	0.54 ± 0.04^a^	0.54 ± 0.03^a^	0.66 ± 0.26^a^
	Heart	0.30 ± 0.01^a,b^	0.29 ± 0.01^a;b^	0.27 ± 0.01^a^	0.32 ± 0.01^a^
	Spleen	0.20 ± 0.02^a^	0.36 ± 0.15^a^	0.26 ± 0.12^a^	0.30 ± 0.08^a^

Male	Liver	3.39 ± 0.44^a^	3.16 ± 0.57^a^	3.23 ± 0.31^a^	3.06 ± 0.15^a^
	Kidneys	0.65 ± 0.05^a^	0.65 ± 0.05^a^	0.65 ± 0.10^a^	0.58 ± 0.04^a^
	Lung	0.59 ± 0.12^a^	0.65 ± 0.05^a^	0.52 ± 0.04^a^	0.53 ± 0.06^a^
	Heart	0.32 ± 0.04^a^	0.31 ± 0.01^a^	0.30 ± 0.01^a^	0.31 ± 0.01^a^
	Spleen	0.35 ± 0.09^b^	0.40 ± 0.09^b^	0.30 ± 0.10^a,b^	0.20 ± 0.01^a^

The table values are presented as mean ± standard deviation of 4 repetitions. In the same line and by sex, the values bearing the different letters are significantly different according to Waller Duncan's multiple comparison test (*p* < 0.05).

**Table 7:** Effect of RVM on biochemical parameters (ALT, AST, total proteins, and alkaline phosphatase) of the rats during subchronic toxicity study.

Sexes	Parameters	Control	Extract doses (mg/kg)		
			250	500	1000
Female	ALT	69.69 ± 1.13^c^	57.88 ± 0.71^a^	66.63 ± 1.43^b^	65.53 ± 0.84^b^
	AST	98.88 ± 2.14^c^	85.31 ± 3.39^b^	74.38 ± 3.27^a^	86.63 ± 1.89^b^
	T. proteins	11.14 ± 0.31^c^	8.80 ± 0.29^a^	9.29 ± 0.46^a,b^	9.90 ± 0.36^b^
	PAL	358.42 ± 7.52^d^	342.46 ± 2.30^c^	324.67 ± 3.94^b^	281.35 ± 4.80^a^

Male	ALT	67.72 ± 1.31^b^	62.47 ± 1.31^a^	69.03 ± 1.80^b^	69.25 ± 2.47^b^
	AST	103.69 ± 5.37^b,c^	87.94 ± 1.52^a^	101.50 ± 5.72^b^	109.38 ± 1.24^c^
	T. proteins	11.31 ± 1.07^b^	9.31 ± 0.82^a^	9.59 ± 1.09^a^	9.95 ± 0.52^a,b^
	PAL	438.22 ± 5.65^d^	417.70 ± 2.98^c^	396.26 ± 4.80^b^	317.83 ± 5.65^a^

The table values are presented as mean ± standard deviation of 4 repetitions. In the same line and by sex, the values bearing the different letters are significantly different according to Waller Duncan's multiple comparison test (*p* < 0.05). Indicators: ALT: alanine aminotransferase, AST: aspartate aminotransaminase; T. proteins: total proteins, ALP: alkaline phosphatase.

**Table 8:** Effect of RVM on the level of serum creatinine, serum urea, and urinary protein.

Sexes	Parameters (mg/dL)	Control	Extract doses (mg/kg)		
			250	500	1000
Females	Serum urea	30.55 ± 1, 15^b^	27.38 ± 1.10^a^	27.15 ± 0.84^a^	25.33 ± 1.78^a^
	Urinary urea	1522.85 ± 12.30^c^	1280.32 ± 8.08^b^	1259.83 ± 5.57^a^	1268.66 ± 6.12^a,b^
	Serum creatinine	0.83 ± 0.05^a,b^	0.83 ± 0.02^a,b^	0.88 ± 0.05^b^	0.80 ± 0.01^a^
	Urinary creatinine	94.51 ± 5.40^c^	55.49 ± 1.22^a^	79.27 ± 1.41^b^	82.93 ± 3.98^b^
	Urinary protein	12.73 ± 1.84^a^	10.74 ± 1.52^a^	12.33 ± 2.72^a^	10.34 ± 0.92^a^

Males	Serum urea	26.07 ± 1.82^a^	24.67 ± 1.40^a^	23.65 ± 1.15^a^	24.58 ± 1.04^a^
	Urinary urea	1318.40 ± 9.24^c^	1235.84 ± 6.10^b^	1184.87 ± 4.80^a^	1231.34 ± 4.45^b^
	Serum creatinine	1.07 ± 0.07^c^	0.83 ± 0.04^b^	0.81 ± 0.02^a,b^	0.73 ± 0.04^a^
	Urinary creatinine	112.20 ± 3.45^b^	95.12 ± 1.99^a^	96.95 ± 4.17^a^	119.51 ± 1.99^c^
	Urinary protein	15.51 ± 1.52^a^	14.72 ± 1.52^a^	14.32 ± 1.84^a^	15.51 ± 0.80^a^

The table values are presented as mean ± standard deviation of 4 repetitions. In the same line and by sex, the values bearing the different letters are significantly different according to Waller Duncan's multiple comparison test (*p* < 0.05).

**Table 9:** Effect of RVM on lipid profile in both sexes of rats during subchronic toxicity study.

Sexes	Parameters (mg/dL)	Control	Extract doses (mg/kg)		
			250	500	1000
Females	TC	80.41 ± 2.31^a^	82.44 ± 3.81^a^	84.73 ± 3.54^a^	84.22 ± 1.47^a^
	HDL	50.95 ± 0.41^a^	52.08 ± 1.67^a^	53.22 ± 1.86^a^	52.46 ± 1.09^a^
	TG	52.00 ± 5.80^a^	50.71 ± 3.83^a^	55.51 ± 6.28^a^	50.26 ± 2.90^a^
	LDL	19.46 ± 1.14^a^	20.62 ± 3.73^a^	20.41 ± 1.90^a^	21.71 ± 2.45^a^

Males	TC	103.82 ± 4.16^a^	104.33 ± 3.30^a^	106.30 ± 3.13^a^	101.78 ± 4.28^a^
	HDL	59.52 ± 1.48^b^	54.60 ± 2.12^a^	54.10 ± 1.99^a^	54.60 ± 4.33^a^
	TG	62.87 ± 4.59^a^	91.18 ± 4.20^c^	92.83 ± 3.67^c^	72.24 ± 3.31^b^
	LDL	31.72 ± 2.43^a^	31.49 ± 1.54^a^	33.63 ± 1.30^a^	32.73 ± 4.65^a^

The table values are presented as mean ± standard deviation of 4 repetitions. In the same line and by sex, the values bearing the different letters are significantly different according to Waller Duncan's multiple comparison test (*p* < 0.05). Indicators: TG: triglyceride; TC: total cholesterol; HDL: high-density lipoproteins; LDL: Low-density lipoproteins.

**Table 10:** Effect of RVM on hematological parameters of the rats treated with RVM during subchronic toxicity study.

Sexes	Parameters	Control	Extract doses (mg/kg)		
			250	500	1000
Females	WBCs (×10^3^ /*µ*l)	4.60 ± 0.36^a^	5.03 ± 0.72^a^	4.25 ± 0.65^a^	7.30 ± 0.87^b^
	Lymph (%)	64.00 ± 4.90^a^	65.93 ± 5.66^a^	63.88 ± 3.63^a^	63.87 ± 3.62^a^
	MONO (%)	6.27 ± 0.40^b^	6.23 ± 0.18^b^	5.75 ± 0.15^b^	4.27 ± 0.97^a^
	GR (%)	29.73 ± 4.51^a^	28.20 ± 1.23^a^	30.45 ± 3.55^a^	31.87 ± 2.26^a^
	PLT (×10^3^/*µ*l)	847.00 ± 7.94^a^	855.00 ± 6.00^a^	850.00 ± 2.00^a^	871.50 ± 5.50^b^
	MPV (fL)	6.77 ± 0.12^a^	6.97 ± 0.31^a^	6.87 ± 0.40^a^	6.90 ± 0.20^a^
	RBCs (×10^6^/*µ*l)	8.88 ± 0.01^a^	8.55 ± 0.15^a^	8.48 ± 0.31^a^	8.62 ± 0.16^a^
	Hb (g/dL)	18.30 ± 0.66^b^	17.47 ± 0.95^a,b^	16.70 ± 0.82^a,b^	16.50 ± 0.79^a^
	HCT (%)	54.43 ± 1.66^b^	51.20 ± 2.07^a,b^	51.17 ± 0.55^a,b^	47.90 ± 3.81^a^
	MCV (fL)	60.10 ± 0.78^a^	59.90 ± 1.56^a^	60.37 ± 2.15^a^	60.83 ± 1.12^a^
	MCH (pg)	20.23 ± 0.93^a^	20.40 ± 0.82^a^	19.70 ± 0.20^a^	21.00 ± 1.04^a^
	MCHC (g/dL)	33.63 ± 1.14^a^	34.33 ± 0.55^a^	32.70 ± 1.55^a^	34.50 ± 1.08^a^

Males	WBCs (×10^3^/*µ*l)	6.20 ± 1.00^a^	6.40 ± 0.26^a^	5.55 ± 1.05^a^	6.35 ± 0.35^a^
	Lymph (%)	69.80 ± 1.91^b^	68.57 ± 3.42^b^	62.30 ± 3.90^b^	51.57 ± 2.43^a^
	MONO (%)	4.40 ± 0.66^a^	4.53 ± 0.55^a^	5.10 ± 0.90^a^	5.47 ± 0.32^a^
	GR (%)	26.80 ± 0.30^a^	26.90 ± 3.60^a^	31.10 ± 1.50^a^	47.97 ± 4.44^b^
	PLT (×10^3^/*µ*l)	666.33 ± 10.97^a^	662.33 ± 4.51^a^	664.00 ± 5.29^a^	860.33 ± 17.04^b^
	MPV (fL)	6.90 ± 0.00^a^	7.43 ± 0.23^a^	7.10 ± 0.20^a^	6.90 ± 1.00^a^
	RBCs (×10^6^/*µ*l)	9.11 ± 0.67^a^	8.67 ± 0.44^a^	9.21 ± 0.65^a^	9.55 ± 0.50^a^
	Hb (g/dL)	17.53 ± 0.12^a^	17.20 ± 0.85^a^	18.05 ± 1.05^a^	17.83 ± 1.47^a^
	HCT (%)	54.57 ± 2.94^a^	53.50 ± 1.00^a^	56.67 ± 1.14^a^	56.63 ± 4.29^a^
	MCV (fL)	59.97 ± 2.03^a^	60.53 ± 0.76^a^	61.70 ± 3.75^a^	59.27 ± 2.57^a^
	MCH (pg)	19.30 ± 1.56^a^	19.87 ± 0.06^a^	20.03 ± 0.76^a^	18.67 ± 0.85^a^
	MCHC (g/dL)	32.17 ± 1.91^a^	32.80 ± 0.35^a^	32.50 ± 0.82^a^	31.50 ± 0.30^a^

The table values are presented as mean ± standard deviation of 4 repetitions. In the same line and by sex, the values bearing the different letters are significantly different according to Waller Duncan's multiple comparison test (*p* < 0.05). Indication: WBCs: white blood cells, Lymph: lymphocytes, Mono: monocytes, GR: granulocytes, PLT: platelets, MPV: mean platelet volume, RBCs: red blood cells, Hb: hemoglobin, HCT: hematocrit, MCV: mean corpuscular volume, MCH: mean corpuscular hemoglobin, MCHC: mean corpuscular hemoglobin concentration.

## Data Availability

All data obtained or generated during this work are incorporated in this published article.

## Supplementary Materials

Supplementary Materials
**S1**: ^1^H NMR (400 MHz DMSO) Spectrum of Compound **1**; **S2**: ^13^C NMR (100 MHz DMSO) Spectrum of Compound **1**; **S3**: ^1^H NMR (600 MHz, CD_3_OD) Spectrum of Compound **2**; **S4**: ^13^C NMR (150 MHz, CD_3_OD) Spectrum of Compound **2**; **S5**: ^1^H NMR (400 MHz CD_3_OD) Spectrum of Compound **3; S6**: ^13^C NMR (100 MHz CD_3_OD) Spectrum of Compound **3**; **S7**: ^1^H NMR (400 MHz CD_3_OD) Spectrum of Compound **4**; **S8**: ^13^C NMR (100 MHz CD_3_OD) Spectrum of Compound **4**; **S9**: ^1^H NMR (400 MHz CDCl_3_) Spectrum of Compound **5; S10**: ^13^C NMR (100 MHz CDCl_3_) Spectrum of Compound **5; S11**: ^1^H NMR (300 MHz DMSO) Spectrum of Compound **6**; **S12**: ^13^C NMR (75 MHz DMSO) Spectrum of Compound **6**; **S13**: ^1^H NMR (400 MHz DMSO) Spectrum of Compound **7**; **S14**: ^13^C NMR (100 MHz DMSO) Spectrum of Compound **7**; **S15**: ^1^H NMR (400 MHz CD_3_OD) Spectrum of Compound **8; S16**: ^13^C NMR (100 MHz CD_3_OD) Spectrum of Compound **8; S17**: ^1^H NMR (400 MHz, CDCl_3_) Spectrum of Compound **9; S18**: ^13^C NMR (100 MHz, CDCl_3_) Spectrum of Compound **9**.
