# Supplementary material for: Botanical from the Fruits Mesocarp of Raphia vinifera Displays Antiproliferative Activity and Is Harmless as Evidenced by Toxicological Assessments
Source: Evid Based Complement Alternat Med. 2022 Mar 29;2022:4831261. doi: 10.1155/2022/4831261 (PMC8983201; doi:10.1155/2022/4831261)
Supplement: Supplementary Materials — S1: 1H NMR (400 MHz DMSO) Spectrum of Compound 1; S2: 13C NMR (100 MHz DMSO) Spectrum of Compound 1; S3: 1H NMR (600 MHz, CD3OD) Spectrum of Compound 2; S4: 13C NMR (150 MHz, CD3OD) Spectrum of Compound 2; S5: 1H NMR (400 MHz CD3OD) Spectrum of Compound 3; S6: 13C NMR (100 MHz CD3OD) Spectrum of Compound 3; S7: 1H NMR (400 MHz CD3OD) Spectrum of Compound 4; S8: 13C NMR (100 MHz CD3OD) Spectrum of Compound 4; S9: 1H NMR (400 MHz CDCl3) Spectrum of Compound 5; S10: 13C NMR (100 MHz CDCl3) Spectrum of Compound 5; S11: 1H NMR (300 MHz DMSO) Spectrum of Compound 6; S12: 13C NMR (75 MHz DMSO) Spectrum of Compound 6; S13: 1H NMR (400 MHz DMSO) Spectrum of Compound 7; S14: 13C NMR (100 MHz DMSO) Spectrum of Compound 7; S15: 1H NMR (400 MHz CD3OD) Spectrum of Compound 8; S16: 13C NMR (100 MHz CD3OD) Spectrum of Compound 8; S17: 1H NMR (400 MHz, CDCl3) Spectrum of Compound 9; S18: 13C NMR (100 MHz, CDCl3) Spectrum of Compound 9. [file 4831261.f1.doc]

**Botanical from the fruits mesocarp of *Raphia vinifera* displays antiproliferative activity and is harmless as evidenced by toxicological assessments**

Gaëlle S. Nguenang^1^, Armelle T. Mbaveng^1^, Idrios N. Bonsou^1^, Godloves F. Chi^2^ and Victor Kuete^1*^

^1^*Department of Biochemistry, Faculty of Science, University of Dschang, Dschang, Cameroon*

^2^*Department of Chemistry, Faculty of Science University of Buea, Buea, Cameroon*

**Corresponding authors:**

*Tel.: +237 677355927; E-mail address:* [*kuetevictor@yahoo.fr*](mailto:kuetevictor@yahoo.fr)*; ORCID: 0000-0002-1070-1236 (Prof. Dr Victor Kuete)*

**Other author’s addresses**

*Gaëlle S. Nguenang:* [*sgaelle78@yahoo.com*](mailto:sgaelle78@yahoo.com)

*Armelle T. Mbaveng:* [*armbatsa@yahoo.fr*](mailto:armbatsa@yahoo.fr)*; ORCID: 0000-0003-4178-4967*

*Idrios N. Bonsou:* [*Bonichrist89@yahoo.com*](mailto:Bonichrist89@yahoo.com )

*Godloves F. Chi:* [*chigodloves@yahoo.com*](mailto:chigodloves@yahoo.com)*; ORCID: 0000-0002-1484-3995*

***SM1. Physical properties and NMR data of Compounds 1 – 9.***

**Compound** **(1)**: (25*R*)-spirost-5-ene-3*β, *22*β*-3-*O*-*β*-_D_-glucopyranosyl(1→2)-*O*-*α*-L-rhamnopyranoside, white solid mp: 209-210 ºC, HRESIMS [M+Na]^+^ m/z 745.4131 (calcd for C_39_H_62_O_12_Na 722.4139).  ^13^C NMR (150 MHz, DMSO); 37.3 (C-1), 29.5 (C-2), 76.4 (C-3), 38.0 (C-4), 140.7 (C-5), 121.7 (C-6), 31.9 (C-7), 31.4 (C-8), 50.0 (C-9), 36.9 (C-10), 20.8 (C-11), 38.0 (C-12), 40.5 (C-13), 56.2 (C-14), 31.4 (C-15), 80.6 (C-16), 62.2 (C-17), 16.5 (C-18), 19.4 (C-19), 41.5 (C-20), 15.2 (C-21), 108.9 (C-22), 31.9 (C-23), 28.9 (C-24), 30.3 (C-25), 66.4 (C-26), 17.6 (C-27), Glucose (C_3_) 98.5 (C-1’), 767 (C-2’), 78.1 (C-3’), 70.6 (C-4’), 77.0 (C-5’), 61.3 (C-6’), Rhamnose (C_1_-C_2_) 100.5 (C-1’’), 70.9 (C-2’’ ), 72.3 (C-3’’), 71.0 (C-4’’), 68.3 (C-5’’), 18.2 (C-6’’).

^1^H NMR (600 MHz, DMSO): 1.80 (1H m, H-1), 1.75 (H, m, H-2), 3.48 (1H, m, H-3), 2.16 (1H, t, J = 11.7 Hz, H-4a), 2.42 (1H, t, J = 11.7, 4.6 Hz, H-4b), 5.34 (1H, d, J = 4.7 Hz, H-6), 1.56 (1H, m, H-7a), 1.30 (1H, m, H-7b) 1.62 (1H, m, H-8), 0.89 (1H, m, H-9), 1.57 (1H, m, H-11), 2.15 (1H, m, H-12a), 1.80 (1H, m, H-12b), 1.15 (1H, m, H-14), 2.01 (1H, m, H-15a), 1.46 (1H, m, H-15b), 4.27 (1H, m, H-16), 1.67 (1H, m, H-17), 0.73 (3H, s, H-18), 0.96 (3H, s, H-19), 1.82 (1H, m, H-20), 0.91 (3H, d, J = 6.8 Hz, H-21), 1.93 (1H, m, H-23a) 1.50 (1H, m, H-23), 1.23 (1H, m, H-24a), 1.25(1H, m, H-24b), 1.75 (1H, m, H-25), 3.41 (1H, m, H-26a), 3.18 (1H, m, H-26b), 0.75 (3H, d, J = 6.4 Hz), Glucose (C1), 4.35 (1H, d, J = 8.0 Hz, 1’), 3.15 (1H, m, H-2’), 3.28 (1H, m, H-3’), 3.02 (1H, m, H-4’), 3.43 (1H, m, H-5’), 3.39 (1H, d, J = 4.0 Hz, H-6a’), 3.66 (1H, d, J = 4.0, H-6b’), Rhamnose (C1-C2), 5.02 (1H, d, J = 1.5 Hz, H-1’’), 3.64 (1H, m, H-2’’), 3.19 (1H, m, H-3’’), 3.41 (1H, m, H-4’’), 4.00 (1H, m, H-5’’), 1.09 (3 H, d, J = 6.0 Hz, H-6’’) [1].

**S1**: ^1^H NMR (400 MHz DMSO) Spectrum of compound **1.**

**S2**: ^13^C NMR (100 MHz DMSO) Spectrum of compound **1.**

**Compound (2)**: **Raphvinin 1 or (25*S*)-26-*O*-(*β*-_D_-galactopyranosyl)-furost-5-ene-3*β*,22*α*,26-trihydroxy-3-*O*-*β*-_D_-glucopyranosyl-(1→2)-*α*-L-rhamnopyranoside**, Cream powder, HRESIMS [M+Na]^+^ m/z 925.4756 (calcd for C_45_H_72_O_17_Na 925.4773).  ^13^C NMR (150 MHz, CD_3_OD); 37.1 (C-1), 29.3 (C-2), 77.7 (C-3), 38.0 (C-4), 140.4 (C-5), 121.2 (C-6), 31.4 (C-7), 31.3 (C-8), 50.2 (C-9), 36.2 (C-10), 20.5 (C-11), 39.4 (C-12), 40.2 (C-13), 56.3 (C-14), 31.3 (C-15), 81.0 (C-16), 63.6 (C-17), 15.4 (C-18), 18.4 (C-19), 39.7 (C-20), 14.7(C-21), 112.5 (C-22), 31.7 (C-23), 27.6 (C-24), 33.3 (C-25), 74.5 (C-26), 15.8 (C-27), Glucose (C_1_) 99.0 (C-1’), 77.6 (C-2’), 76.3 (C-3’), 70.2 (C-4’), 77.9 (C-5’), 61.4 (C-6’), Rhamnose (C_1_-C_2_) 100.7 (C-1’’), 70.4 (C-2’’ ), 70.9 (C-3’’), 72.3 (C-4’’), 68.2 (C-5’’), 18.2 (C-6’’), Galactose (C_26_) 103.1 (C-1’), 73.7 (C-2’), 70.3 (C-3’), 69.8 (C-4’), 7.4 (C-5’), 61.3 (C-6’). ^1^H NMR (600 MHz, CD_3_OD): 1.72, 0.92 (2H m, H-1a, H-1b), 1.75, 1.38 (2H, m, H-2a, H-2b), 3.44 (1H, m, H-3), 2.32 (1H, t, J = 11.7 Hz, H-4a), 2.09 (1H, t, J = 11.7, 4.6 Hz, H-4b), 5.28 (1H, d, J = 4.7 Hz, H-6), 1.82 (1H, m, H-7a), 1.07 (1H, m, H-7b) 1.47 (1H, m, H-8), 0.82 (1H, m, H-9), 1.41 (1H, m, H-11), 1.63 (1H, m, H-12a), 1.06 (1H, m, H-12b), 1.01 (1H, m, H-14), 1.88 (1H, m, H-15a), 1.46 (1H, m, H-15b), 4.38 (1H, m, H-16), 1.61 (1H, m, H-17), 0.67 (3H, s, H-18), 0.89 (3H, s, H-19), 1.89 (1H, m, H-20), 0.85 (3H, d, J = 6.6 Hz, H-21), 1.88 (1H, m, H-23a) 1.50 (1H, m, H-23b), 1.43 (1H, m, H-24a), 1.25(1H, m, H-24b), 1.58 (1H, m, H-25), 3.48 (1H, m, H-26a), 3.25 (1H, m, H-26b), 0.76 (3H, d, J = 6.3 Hz), Glucose (C3), 4.49 (1H, d, J = 3.4 Hz, 1’), 3.38 (1H, m, H-2’), 3.10 (1H, m, H-3’), 3.27 (1H, m, H-4’), 3.48 (1H, m, H-5’), 3.67 (1H, d, J = 4.0 Hz, H-6a’), 3.87 (1H, d, J = 4.0, H-6b’), Rhamnose (C1-C2), 5.20 (1H, d, J = 2.4 Hz, H-1’’), 3.93 (1H, m, H-2’’), 3.68 (1H, m, H-3’’), 3.40 (1H, m, H-4’’), 4.16 (1H, m, H-5’’), 1.25 (3 H, d, J = 6.0 Hz, H-6’’), Galactose (C26), 4.24 (1H, d, J = 11.4 Hz, 1’), 3.20 (1H, m, H-2’), 3.27 (1H, m, H-3’), 3.28 (1H, m, H-4’), 3.36 (1H, m, H-5’), 3.64 (1H, d, J = 4.0 Hz, H-6a’), 3.84 (1H, d, J = 4.0, H-6b’) [1].

**S3**: ^1^H NMR (600 MHz, CD_3_OD) Spectrum of Compound **2.**

**S4**: ^13^C NMR (150 MHz, CD_3_OD) Spectrum of Compound **2.**

**Compound (3)**: **Raphvinin 2 or (25*R*)-26-*O*-(*β*-D-galactopyranosyl)-furost-5-ene-3*β*, 22*α*, 26−trihydroxy-3-*O*-*β*-_D_-glucopyranosyl-(1→2)-[*α*-L-rhamnopyranosyl-(1→3)]-*β*-_D_-glucopyranoside,** cream powder, HRESIMS [M+Na]^+^ m/z 1087.528 (calcd for C_51_H_84_O_23_Na 1087.5301).  ^13^C NMR (150 MHz, CD_3_OD); 37.1 (C-1), 29.3 (C-2), 77.9 (C-3), 38.0 (C-4), 140.4 (C-5), 121.1 (C-6), 31.3 (C-7), 29.9 (C-8), 50.2 (C-9), 36.6 (C-10), 20.7 (C-11), 39.7 (C-12), 40.4 (C-13), 56.3 (C-14), 31.7 (C-15), 81.0 (C-16), 63.6 (C-17), 15.4 (C-18), 18.4 (C-19), 40.0 (C-20), 14.7(C-21), 112.5 (C-22), 31.3 (C-23), 27.5 (C-24), 33.5 (C-25), 74.8 (C-26), 15.9 (C-27), Glucose (C_1_) 99.0 (C-1’), 77.9 (C-2’), 78.3 (C-3’), 70.2 (C-4’), 77.7 (C-5’), 61.3 (C-6’), Glucose (C_2_) 101.1 (C-1’), 76.3 (C-2’), 76.4 (C-3’), 70.1 (C-4’), 76.7 (C-5’), 63.5 (C-6’), Rhamnose (C_1_-C_2_) 100.7 (C-1’’), 70.9 (C-2’’ ), 70.7 (C-3’’), 72.5 (C-4’’), 68.3 (C-5’’), 16.5 (C-6’’), Galactose (C_26_) 103.2 (C-1’), 73.7 (C-2’), 76.5 (C-3’), 70.2 (C-4’), 76.7 (C-5’), 61.3 (C-6’). ^1^H NMR (600 MHz, CD_3_OD): 1.88 (2H m, H-1a, H-1b), 1.74, 1.30 (2H, m, H-2a, H-2b), 3.59 (1H, m, H-3), 2.28 (1H, m, H-4a), 2.43 (1H, m, H-4b), 5.40 (1H, *br* s, H-6), 1.98 (1H, m, H-7a), 1.28 (1H, m, H-7b) 1.92 (1H, m, H-8), 0.98 (1H, m, H-9), 1.56 (1H, m, H-11), 1.78 (1H, m, H-12a), 1.18 (1H, m, H-12b), 1.13 (1H, m, H-14), 1.98 (1H, m, H-15a), 1.46 (1H, m, H-15b), 4.39 (1H, m, H-16), 1.74 (1H, m, H-17), 0.85 (3H, s, H-18), 1.06 (3H, s, H-19), 2.18 (1H, m, H-20), 1.03 (3H, d, J = 6.8 Hz, H-21), 1.65 (1H, m, H-23a) 1.50 (1H, m, H-23b), 1.14 (1H, m, H-24a), 1.25(1H, m, H-24b), 1.76 (1H, m, H-25), 3.72 (1H, m, H-26a), 3.42 (1H, m, H-26b), 0.85 (3H, d, J = 4.4 Hz), Glucose (C1), 4.49 (1H, d, J = 8.0 Hz, 1’), 3.48 (1H, m, H-2’), 3.59 (1H, m, H-3’), 3.80 (1H, m, H-4’), 3.37 (1H, m, H-5’), 3.85 (1H, m, H-6a’), 3.66 (1H, m, H-6b’), Glucose (C2), 4.44 (1H, d, J = 7.9 Hz, 1’), 3.24 (1H, m, H-2’), 3.28 (1H, m, H-3’), 3.80 (1H, m, H-4’), 3.38 (1H, m, H-5’), 3.85 (1H, m, H-6a’), 3.68 (1H, m, H-6b’), Rhamnose (C1-C2), 5.21 (1H, *br* s, H-1’’), 3.92 (1H, m, H-2’’), 4.02 (1H, m, H-3’’), 3.40 (1H, m, H-4’’), 4.15 (1H, m, H-5’’), 1.25 (3 H, d, J = 6.0 Hz, H-6’’), Galactose (C26), 4.27 (1H, d, J = 7.9 Hz, 1’), 3.19 (1H, m, H-2’), 3.47 (1H, m, H-3’), 3.80 (1H, m, H-4’), 3.35 (1H, m, H-5’), 3.67 (1H, d, J = 4.0 Hz, H-6a’), 3.95 (1H, d, J = 4.0, H-6b’) [1].

**S5**: ^1^H NMR (400 MHz CD_3_OD) Spectrum of Compound **3.**

**S6**: ^13^C NMR (100 MHz CD_3_OD) Spectrum of Compound **3.**

**Compound (4)**: **Raphvinin 3 or (25*R*)-26-*O*-[*β*-_D_-glucopyranosyl-(1→4)]-*β*-_D_-galactopyranosyl)-furost-5-ene-3*β*, 26-dihydroxy-22*α*-methoxy-3-*O*-*β*-D-glucopyranosyl-(1→2)-[*α*-L-rhamnopyranosyl-(1→2)]-*β*-_D_-lucopyranosyl-(1→4)-*β*-D-glucopyranoside,** transparent crystalline solid, HRESIMS [M+Na]^+^ m/z 1209.6292 (calcd for C_57_H_94_O_28_Na 1209.5904).  ^13^C NMR (150 MHz, CD_3_OD); 37.1 (C-1), 29.3 (C-2), 77.7 (C-3), 38.0 (C-4), 140.5 (C-5), 121.2 (C-6), 31.3 (C-7), 33.5 (C-8), 50.2 (C-9), 36.6 (C-10), 20.5 (C-11), 39.7 (C-12), 40.4 (C-13), 56.3 (C-14), 31.7 (C-15), 81.0 (C-16), 63.6 (C-17), 15.4 (C-18), 18.4 (C-19), 40.4 (C-20), 14.7 (C-21), 112.5 (C-22), 31.3 (C-23), 27.5 (C-24), 33.5 (C-25), 74.5 (C-26), 15.9 (C-27), 46.2 (OCH_3_), Glucose (C_1_) 99.0 (C-1’), 77.6 (C-2’), 77.9 (C-3’), 73.7 (C-4’), 76.5 (C-5’), 61.3 (C-6’), Rhamnose (C_1_-C_2_) 100.7 (C-1’’), 70.7 (C-2’’ ), 70.9 (C-3’’), 72.5 (C-4’’), 68.3 (C-5’’), 16.5 (C-6’’), Glucose (C_2_) 96.7 (C-1’), 74.8 (C-2’), 70.2 (C-3’), 76.4 (C-4’), 76.6 (C-5’), 61.3 (C-6’), Galactose (C_26_) 103.1 (C-1’), 76.6 (C-2’), 76.6 (C-3’), 71.5 (C-4’), 76.6 (C-5’), 61.4 (C-6’). Glucose (C_3_) 92.5 (C-1’), 72.4 (C-2’), 73.4 (C-3’), 70.5 (C-4’), 76.3 (C-5’), 61.3 (C -6’). ^1^H NMR (600 MHz, CD_3_OD): 1.90 (2H m, H-1a, H-1b), 1.93, 1.62 (2H, m, H-2a, H-2b), 3.65 (1H, m, H-3), 2.33 (1H, m, H-4a), 2.46 (1H, m, H-4b), 5.40 (1H, *br* s, H-6), 1.68 (1H, m, H-7a), 1.32 (1H, m, H-7b) 1.77 (1H, m, H-8), 0.97 (1H, m, H-9), 1.58 (1H, m, H-11), 2.19 (1H, t, 6.6 Hz, H-12a), 1.80 (1H, m, H-12b), 1.11 (1H, m, H-14), 1.58 (1H, m, H-15a), 1.46 (1H, m, H-15b), 4.39 (1H, dd, 6.0, 1.8 Hz, H-16), 1.75 (1H, m, H-17), 0.85 (3H, s, H-18), 1.07 (3H, s, H-19), 2.19 (1H, m, H-20), 1.03 (3H, d, J = 7.2 Hz, H-21), 1.68 (1H, m, H-23a) 1.20 (1H, m, H-23b), 1.77 (1H, m, H-24a), 1.25(1H, m, H-24b), 1.76 (1H, m, H-25), 3.77 (1H, m, H-26a), 3.41 (1H, m, H-26b), 0.96 (3H, d, J = 7.2 Hz), 3.16 (3H, s, OCH_3_), Glucose (C1), 4.50 (1H, d, J = 7.8 Hz, H-1’), 3.37 (1H, m, H-2’), 3.48 (1H, m, H-3’), 3.20 (1H, m, H-4’), 3.62 (1H, m, H-5’), 3.79 (1H, m, H-6a’), 3.66 (1H, m, H-6b’), Rhamnose (C1-C2), 5.21 (1H, *br* s, H-1’’), 3.93 (1H, m, H-2’’), 3.67 (1H, m, H-3’’), 3.41 (1H, m, H-4’’), 4.16 (1H, m, H-5’’), 1.26 (3 H, d, J = 5.4 Hz, H-6’’), Glucose (C2), 4.49 (1H, d, J = 7.8 Hz, H-1’), 3.14 (1H, m, H-2’), 3.36 (1H, m, H-3’), 3.35 (1H, m, H-4’), 3.32 (1H, m, H-5’), 3.87 (1H, m, H-6a’), 3.67 (1H, m, H-6b’), Galactose (C26), 4.26 (1H, d, J = 8.4 Hz, H-1’), 3.21 (1H, m, H-2’), 3.26 (1H, m, H-3’), 3.79 (1H, m, H-4’), 3.30 (1H, m, H-5’), 3.88 (1H, d, J = 4.0 Hz, H-6a’), 3.66 (1H, d, J = 4.0, H-6b’), Glucose (C3), 5.12 (1H, d, J = 4.8 Hz, H-1’), 3.37 (1H, m, H-2’), 3.68 (1H, m, H-3’), 3.49 (1H, m, H-4’), 3.30 (1H, m, H-5’), 3.86 (1H, m, H-6a’), 3.67 (1H, m, H-6b’) [1].

**S7**: ^1^H NMR (400 MHz CD_3_OD) Spectrum of Compound **4.**

**S8**: ^13^C NMR (100 MHz CD_3_OD) Spectrum of Compound **4.**

**Compound (5)**: **Diosgenin,** white powder soluble in CHCl_3_, HRESIMS [M+H]^+^ m/z 415.3222 (calcd for C_27_H_43_ O_3_ 415.3212).  ^13^C NMR (100 MHz, CDCl_3_); 37.2 (C-1), 27.1 (C-2), 71.8 (C-3), 39.8 (C-4), 140.8 (C-5), 121.5 (C-6), 31.4 (C-7), 32.0 (C-8), 50.0 (C-9), 36.6 (C-10), 20.9 (C-11), 39.5 (C-12), 40.3 (C-13), 56.5 (C-14), 31.8 (C-15), 80.9 (C-16), 61.9 (C-17), 16.3 (C-18), 19.4 (C-19), 42.3 (C-20), 14.4 (C-21), 109.8 (C-22), 31.6 (C-23), 28.9 (C-24), 31.4 (C-25), 66.1 (C-26), 16.3 (C-27), ^1^H NMR (400 MHz, CDCl_3_): 1.73, 0.96 (2H m, H-1), 1.78, 1.55 (2H, m, H-2), 3.54 (1H, m, H-3), 2.33 (1H, t, J = 2.0 Hz, H-4a), 2.27 (1H, t, J = 2.0 Hz, H-4b), 5.37 (1H, t, J = 2.0 Hz, H-6), 1.55 (1H, m, H-7a), 1.28 (1H, m, H-7b) 1.50 (1H, m, H-8), 0.87 (1H, m, H-9), 1.46, 0.88 (1H, m, H-11), 1.54 (1H, m, H-12a), 1.20 (1H, m, H-12b), 1.15 (1H, m, H-14), 1.75 (1H, m, H-15a), 1.46 (1H, m, H-15b), 4.44 (1H, q, J = 5.2, Hz, H-16), 1.67 (1H, m, H-17), 0.81 (3H, s, H-18), 1.05 (3H, s, H-19), 1.82 (1H, m, H-20), 1.11 (3H, d, J = 4.4 Hz, H-21), 1.93 (1H, m, H-23a), 1.53 (1H, m, H-23), 1.49 (1H, m, H-24a), 1.46 (1H, m, H-24b), 1.84 (1H, m, H-25), 3.98 (1H, dd, J = 2.0, 7.2 Hz, H-26a), 3.33 (1H, d, J =7.6 Hz H-26b), 1.03 (3H, d, J = 4.4 Hz, H-27) [2].

**S9**: ^1^H NMR (400 MHz CDCl_3_) Spectrum of Compound **5.**

**S10**: ^13^C NMR (100 MHz CDCl_3_) Spectrum of Compound **5.**

**Compound (6): Trillin or Diosgenin-3-*O*-*β*-_D_-glucopyranoside or (22*R*, 25*R*)-3*β*-spirost-5-ene-3-O-*β*-_D_-glucopyranoside,** transparent crystals, HRESIMS [M+Na]^+^ m/z 599.3560 (calcd for C_33_H_52_ NaO_8_ 599.3560).  ^13^C NMR (125 MHz, DMSO); 37.2 (C-1), 29.7 (C-2), 77.2 (C-3), 38.7 (C-4), 140.9 (C-5), 121.4 (C-6), 31.4 (C-7), 31.9 (C-8), 50.0 (C-9), 36.8 (C-10), 20.1 (C-11), 39.5 (C-12), 40.2 (C-13), 56.2 (C-14), 31.4 (C-15), 80.6 (C-16), 62.2 (C-17), 16.3 (C-18), 19.5 (C-19), 42.3 (C-20), 15.1 (C-21), 108.8 (C-22), 31.3 (C-23), 28.9 (C-24), 30.2 (C-25), 66.3 (C-26), 17.5 (C-27), Glucose (C_3_) 101.2 (C-1’), 73.9 (C-2’), 77.0 (C-3’), 70.9 (C-4’), 76.7 (C-5’), 61.2 (C-6’). ^1^H NMR (500 MHz, DMSO): 1.73, 0.96 (2H m, H-1), 1.78, 1.55 (2H, m, H-2), 3.59 (1H, m, H-3), 2.39 (1H, t, J = 13.0 Hz, H-4a), 2.12 (1H, t, J = 12.0 Hz, H-4b), 5.32 (1H, dd, J = 2.0, 5.5 Hz, H-6), 1.55 (1H, m, H-7a), 1.28 (1H, m, H-7b) 1.50 (1H, m, H-8), 0.87 (1H, m, H-9), 1.46, 0.88 (1H, m, H-11), 1.54 (1H, m, H-12a), 1.20 (1H, m, H-12b), 1.15 (1H, m, H-14), 1.75 (1H, m, H-15a), 1.46 (1H, m, H-15b), 4.43 (1H, t, J = 5.7, Hz, H-16), 1.67 (1H, m, H-17), 0.74 (3H, s, H-18), 0.97 (3H, s, H-19), 1.82 (1H, m, H-20), 0.92 (3H, d, J = 6.5 Hz, H-21), 1.93 (1H, m, H-23a), 1.53 (1H, m, H-23), 1.49 (1H, m, H-24a), 1.46 (1H, m, H-24b), 1.84 (1H, m, H-25), 3.41 (1H, m, H-26a), 3.20 (1H, m, H-26b), 0.74 (3H, d, J = 8.5 Hz, H-27), Glucose (C3), 4.22 (1H, d, J = 7.8 Hz, H-1’), 2.91 (1H, m, H-2’), 3.05 (1H, m, H-3’), 3.09 (1H, m, H-4’), 3.14 (1H, m, H-5’), 3.65 (1H, d, J = 6.0 Hz, H-6a’), 3.41 (1H, d, J = 5.4 Hz, H-6b’) [3].

**S11**: ^1^H NMR (300 MHz DMSO) Spectrum of Compound **6.**

**S12**: ^13^C NMR (75 MHz DMSO) Spectrum of Compound **6.**

**Compound (7)**: **Deltonin** white powder, HRESIMS [M+H]^+^ m/z 885.4848 (calcd for C_45_H_72_O_17_ 884.4770).  ^13^C NMR (125 MHz, MeOD); 37.1 (C-1), 29.3 (C-2), 77.6 (C-3), 38.7 (C-4), 140.6 (C-5), 121.9 (C-6), 32.1 (C-7), 31.2 (C-8), 49.9 (C-9), 36.2 (C-10), 20.8 (C-11), 39.6 (C-12), 40.5 (C-13), 56.2 (C-14), 31.4 (C-15), 80.5 (C-16), 61.7 (C-17), 16.0 (C-18), 18.9 (C-19), 41.4 (C-20), 16.2 (C-21), 110.4 (C-22), 31.4 (C-23), 27.8 (C-24), 29.7 (C-25), 62.8 (C-26), 16.5 (C-27), Glucose (C_3_) 98.6 (C-1’), 77.6 (C-2’), 74.5 (C-3’), 70.2 (C-4’), 76.7 (C-5’), 61.3 (C-6’), Rhamnose (C_1_-C_2_) 100.6 (C-1’’), 70.9 (C-2’’ ), 72.1 (C-3’’), 70.8 (C-4’’), 68.5 (C-5’’), 17.2 (C-6’’), Glucose (C_3_) 102.9 (C-1’), 73.7 (C-2’), 76.4 (C-3’), 70.6 (C-4’), 77.0 (C-5’), 61.2 (C-6’). ^1^H NMR (500 MHz, MeOD): 1.73 (1H m, H-1), 1.71 (H, m, H-2), 3.12 (1H, m, H-3), 2.22 (1H, t, J = 6.0 Hz, H-4a), 2.10 (1H, t, J = 12.5 Hz, H-4b), 5.28 (1H, *br* s, H-6), 1.80 (1H, m, H-7a), 1.35 (1H, m, H-7b) 1.46 (1H, m, H-8), 0.79 (1H, m, H-9), 1.33 (1H, m, H-11), 1.55 (1H, m, H-12a), 1.01 (1H, m, H-12b), 0.94 (1H, m, H-14), 1.80 (1H, m, H-15a), 1.46 (1H, m, H-15b), 4.38 (1H, dd J = 7.5, 1.5 Hz, H-16), 1.60 (1H, m, H-17), 0.61 (3H, s, H-18), 0.84 (3H, s, H-19), 1.71 (1H, m, H-20), 0.79 (3H, d, J = 6.5 Hz, H-21), 1.62 (1H, m, H-23a) 1.50 1H, m, H-23), 1.40 (1H, m, H-24a), 1.25(1H, m, H-24b), 1.52 (1H, m, H-25), 3.36 (1H, m, H-26a), 3.36 (1H, m, H-26b), 0.62 (3H, d, J = 8.5 Hz), Glucose (C3), 4.32 (1H, d, J = 7.5 Hz, H-1’), 3.13 (1H, m, H-2’), 3.32 (1H, m, H-3’), 3.20 (1H, m, H-4’), 3.22 (1H, m, H-5’), 3.43 (1H, d, J = 4.0 Hz, H-6a’), 3.63 (1H, d, J = 4.0, H-6b’), Rhamnose (C1-C2), 4.95 (1H, *br* s, H-1’’), 3.75 (1H, m, H-2’’), 3.22 (1H, m, H-3’’), 3.78 (1H, m, H-4’’), 3.93 (1H, d, J = 6.0 Hz, H-5’’), 1.09 (3 H, d, J = 1.5, 7.5, H-6’’) Glucose (C3), 4.09 (1H, d, J = 7.5 Hz, 1’), 3.49 (1H, m, H-2’), 3.32 (1H, m, H-3’), 3.20 (1H, m, H-4’), 3.22 (1H, m, H-5’), 3.55 (1H, d, J = 4.0 Hz, H-6a’), 3.66 (1H, d, J = 4.0, H-6b’) [4].

**S13**: ^1^H NMR (400 MHz DMSO) Spectrum of Compound **7.**

**S14**: ^13^C NMR (100 MHz DMSO) Spectrum of Compound **7.**

**Compound (8)**: 26-O-*β*-_D_-glucopyranosyl-(22*R*,25*R*)-3*β*, 22, 26-trihydroxyfurost-5-ene-3-O-*β*-_D_-glucopyranoside, white powder, HRESIMS [M+Na]^+^ m/z 925.4756 (calcd for C_45_H_72_O_17_Na 925.4773).  ^13^C NMR (125 MHz, CD_3_OD); 37.1 (C-1), 29.3 (C-2), 78.4 (C-3), 38.3 (C-4), 140.6 (C-5), 121.1 (C-6), 31.4 (C-7), 31.8 (C-8), 50.3 (C-9), 36.6 (C-10), 20.6 (C-11), 39.4 (C-12), 40.4 (C-13), 56.3 (C-14), 31.4 (C-15), 81.0 (C-16), 63.7 (C-17), 15.4 (C-18), 18.5 (C-19), 39.8 (C-20), 14.8 (C-21), 112.5 (C-22), 31.7 (C-23), 27.6 (C-24), 33.6 (C-25), 74.5 (C-26), 15.9 (C-27), Glucose (C_3_) 101.1 (C-1’), 76.7 (C-2’), 76.4 (C-3’), 70.2 (C-4’), 76. (C-5’), 61.3 (C-6’), Glucose (C_26_) 103.2 (C-1’), 76.6 (C-2’), 76.5 (C-3’), 70.3 (C-4’), 76.5 (C-5’), 61.4 (C-6’). ^1^H NMR (500 MHz, CD_3_OD): 1.79, 1.01 (2H m, H-1a, H-1b), 1.89, 1.46 (2H, m, H-2a, H-2b), 3.49 (1H, m, H-3), 2.32 (1H, t, J = 11.7 Hz, H-4a), 2.09 (1H, t, J = 11.7, 4.6 Hz, H-4b), 5.28 (1H, d, J = 4.7 Hz, H-6), 1.82 (1H, m, H-7a), 1.07 (1H, m, H-7b) 1.47 (1H, m, H-8), 0.88 (1H, m, H-9), 1.46 (1H, m, H-11), 1.69 (1H, m, H-12a), 1.09 (1H, m, H-12b), 1.04 (1H, m, H-14), 1.89 (1H, m, H-15a), 1.19 (1H, m, H-15b), 4.38 (1H, m, H-16), 1.61 (1H, m, H-17), 0.74 (3H, s, H-18), 0.95 (3H, s, H-19), 2.08 (1H, m, H-20), 0.85 (3H, d, J = 6.6 Hz, H-21), 1.88 (1H, m, H-23a) 1.50 (1H, m, H-23b), 1.50 (1H, m, H-24a), 1.06 (1H, m, H-24b), 1.58 (1H, m, H-25), 3.48 (1H, m, H-26a), 3.25 (1H, m, H-26b), 0.91 (3H, d, J = 6.3 Hz, H-27), Glucose (C3), 4.28 (1H, d, J = 3.4 Hz, H-1’), 3.38 (1H, m, H-2’), 3.10 (1H, m, H-3’), 3.28 (1H, m, H-4’), 3.48 (1H, m, H-5’), 3.67 (1H, d, J = 4.0 Hz, H-6a’), 3.87 (1H, d, J = 4.0, H-6b’), Glucose (C26), 4.13 (1H, d, J = 3.4 Hz, 1’), 3.38 (1H, m, H-2’), 3.16 (1H, m, H-3’), 3.23 (1H, m, H-4’), 3.48 (1H, m, H-5’), 3.57 (1H, d, J = 4.0 Hz, H-6a’), 3.76 (1H, d, J = 4.0, H-6b’), [3].

**S15**: ^1^H NMR (400 MHz CD_3_OD) Spectrum of Compound **8.**

**S16**: ^13^C NMR (100 MHz CD_3_OD) Spectrum of Compound **8.**

Compound (**9**): Stigmasterol or stigmasta-5,22(*E*)-dien-3*α*-ol (C_29_H_48_O); white solid soluble in CDCl_3_,^1^H and ^13^C NMR data: ESI-MS [M+H] peak at *m/z* 413.8. mp: 170-171^o^C. PubChem CID: 5280794.

^1^H NMR (400 MHz, CDCl_3_), *δ* (ppm): 5.37 (1H, q, *J* = 3.6, 2.4 Hz, H-6), 5.16 (1H, q, *J* = 9.0, 3.0 Hz, H-22), 5.04 (1H, q, *J* = 9.0, 6.0 Hz, H-23), 3.55 (1H, m, H-3), 1.04 (3H, t, H-27), 1.03 (3H, d, *J* = 6.6 Hz, H-21), 0.94 (3H, d, H-28), 0.86 (3H, d, *J* = 6.5 Hz, H-26), 0.82 (3H, t, *J* = 6.0 Hz, H-29), 0.82 (3H, d, *J* = 6.0 Hz, H-19), 0.71 (3H, s, H-18). ^13^C NMR (100 MHz, CDCl_3_), *δ* (ppm):140.7 (C-5), 138.3 (C-22), 129.3 (C-23), 121.8 (C-6), 71.8 (C-3), 56.9 (C-14), 55.9 (C-17), 51.1 (C-24), 50.1 (C-9), 42.2 (C-4), 42.2 (C-13), 40.5 (C-20), 39.6 (C-12), 37.1 (C-1), 36.5 (C-10), 31.9 (C-25), 31.8 (C-7), 31.6 (C-8), 29.7 (C-2), 28.9 (C-16), 25.4 (C-28), 24.3 (C-15), 21.2 (C-19), 21.1 (C-11), 21.0 (C-26), 19.4 (C-27), 18.9 (C-28), 12.2 (C-29), 12.0 (C-18) [5], [6].

**S17**: ^1^H NMR (400 MHz, CDCl_3_) Spectrum of Compound **9.**

**S18**: ^13^C NMR (100 MHz, CDCl_3_) Spectrum of Compound **9.**

**References:**

[1] G. F. Chi, R. V. T. Sop, A. T. Mbaveng, and J. O. Ombito, “Steroidal saponins from Raphia vinifera and their cytotoxic activity,” *Steroids*, vol. 163, no. July, pp. 108724, 2020.

[2] K. Pazhanichamy, K. Bhuvaneswari, B. Kunthavai, and T. Eevera, “Isolation , Characterization and Quantification of Diosgenin from Costus igneus,” *J. Planar Chromatogr.*, vol. 25, no. October, pp. 6–13, 2012.

[3] M. Yoshikawa *et al.*, “Medicinal Flowers. XII.1) New Spirostane-Type Steroid Saponins with Antidiabetogenic Activity from Borassus flabellifer,” *Chem. Pharm. Bull. (Tokyo).*, vol. 55, no. 2, pp. 308–316, 2007.

[4] W. K. and J. J. D. V. Patricia Y. Hayes, Lynette K. Lambert, Reg Lehmann, Kerry Penman, “Complete 1H and 13C assignments of the four major saponins from Dioscorea villosa (wild yam),” *Magn. Reson. Chem.*, vol. 45, no. December 2006, pp. 185–188, 2007.

[5] M. Arora and A. N. Kalia, “Isolation and characterization of stigmasterol and β-sitosterol-d-glycoside from ethanolic extract of the stems of salvadora persica Linn.,” *Int. J. Pharm. Pharm. Sci.*, vol. 5, pp. 1–5, 2013.

[6] V. Sai, P. Chaturvedula, and I. Prakash, “Isolation of stigmasterol and β -sitosterol from the dichloromethane extract of *Rubus suavissimus*,” vol. 1, no. 9, pp. 239–242, 2012.
